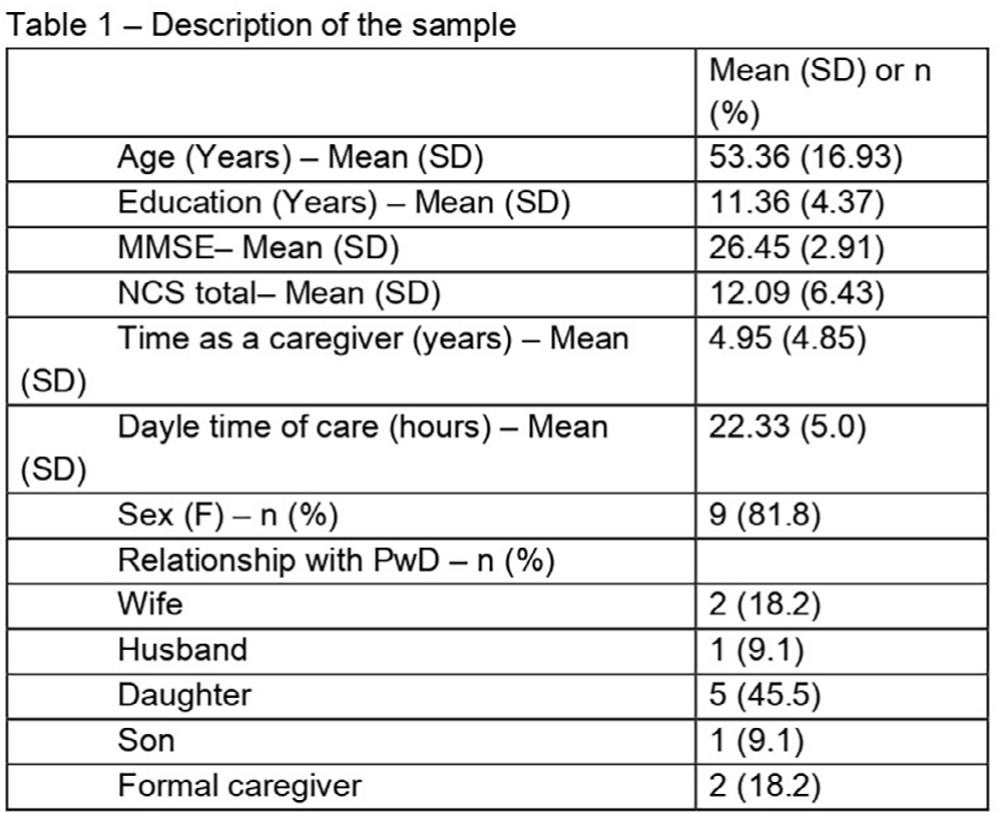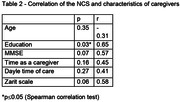# Investigation of correlation between negative communication and characteristics of caregivers of people with dedmentia

**DOI:** 10.1002/alz.093040

**Published:** 2025-01-09

**Authors:** Amanda Gorziza da Silva, Lucimara Lehmen Gheno, Liana Lisboa Fernandez, Carlos Roberto Mello Rieder, Bárbara Costa Beber

**Affiliations:** ^1^ Federal University of Health Sciences of Porto Alegre (UFCSPA), Viamão, Rio Grande do Sul Brazil; ^2^ UFCSPA, porto alegre, Rio Grande do Sul Brazil; ^3^ Universidade Federal de Ciências da Saúde de Porto Alegre, Porto Alegre, Rio Grande do Sul Brazil; ^4^ Federal University of Health Sciences of Porto Alegre (UFCSPA), Porto Alegre, Rio Grande do Sul Brazil; ^5^ Universidade Federal de Ciências da Saúde de Porto Alegre (UFCSPA), Porto Alegre, RS Brazil

## Abstract

**Background:**

Caregivers of people with dementia (PwD), assuming the responsibility for another life, can become physically and emotionally overwhelmed, leading to increased psychiatric symptoms such as anxiety and depression. Similarly, the level of neuropsychiatric symptoms in PwD is associated with caregiver burden. Faced with this burden and the challenges of managing dementia, caregivers may potentially channel their feelings of frustration and exhaustion from caregiving into the way they communicate with the person with dementia. It is important to identify the determinants of negative communication by caregivers to develop educational programs to improve communication. The objective of this study was to verify the correlation between negative communication and characteristics of caregivers of PwD.

**Method:**

This is an excerpt from a descriptive cross‐sectional observational study involving a sample of 11 caregivers. We collected sociodemographic data and information regarding the characteristics of care. The following tools were administered to caregivers of PwD: Negative Communication Scale (NCS), which assesses the caregiver’s negative communication through questions related to daily communication with the patient; Zarit Burden Scale, which assesses caregiver burden; Mini‐Mental State Examination (MMSE), a screening test for a quick evaluation of cognitive function. Statistical analyses were conducted using Chi‐Square and Spearman tests with a significance level of 5%.

**Result:**

The study included 11 caregivers of PwD. Their characteristics are presented in Table 1. Table 2 shows the correlations between the NCS and characteristics of the sample. There was a positive correlation between the NCS and education, suggesting that the higher the education, the worse the perception of the caregivers regarding their use of negative communication. Despite the absence of statistical significance, there was a trend for positive correlations with MMSE and Zarit scores. Additionally, there was no significant difference in the NCS when compared between sexes (p = 0.57) and types of relationships of the caregivers with the PwD (p = 0.48) (Chi‐Square test).

**Conclusion:**

Education may be correlated with the occurrence or perception of negative communication acts used by caregivers. Despite the presented correlations, it is necessary to increase the sample size to demonstrate/confirm these correlations, as well as trends in correlations.